# ‘They attacked you just like that’: Negotiating racial epistemics in making claims about racism

**DOI:** 10.1111/bjso.12846

**Published:** 2025-01-11

**Authors:** Rahul Sambaraju

**Affiliations:** ^1^ Department of Psychology The University of Edinburgh Edinburgh UK

**Keywords:** African, Black, discursive psychology, epistemics, India, prejudice, racism

## Abstract

Social psychological research on race and racism has shown that claims about racism are not always accepted or received as valid reports. In this paper, I offer racial epistemics as one mechanism by which race‐talk takes place. I examine how ascribing category‐bound entitlements to experiential or other knowledge about racism is variously realised and complicated in the production of claims about racism. Through examining news media accounts where Black persons were invited to talk about their experiences of racism in India, I show that despite ascribing a privileged epistemic position to Black persons, recipients (interviewers and other panellists) could make salient epistemic entitlements to commonsense, specialised, or other forms of (racial) knowledge in collaboratively establishing, confirming or correcting, and challenging claims about racism in India. The findings are discussed in relation to the broader understanding of racism in social psychology. The data are in Indian English.

## INTRODUCTION

Social psychologists and race scholars show that those who are targets of racism face the problem of having their reports of racist experiences discounted, denied, or undermined (Augoustinos & Every, 2007; Bonilla‐Silva, 1997; Essed, 1991b; Goodman, 2014; Van Dijk, 1992). Individuals face concerns about identifying and reporting whether an instance is indeed racist–the concern ostensibly is with clarity on what is to count as racism. In this paper, I show that differential rights and entitlements to knowing racism–racial epistemics–are one set of resources implicated in the telling and denials of racism. I do this in a post‐colonial setting, where both social groups are associated with colonial and racial oppression and which is barely the subject of serious examination in social psychology: anti‐Black racism in India (Sambaraju, 2021).

Discursive psychological research in Euro‐American and other settler colonial settings has shown that race, racism, and related terms (like racial categories or migrant and non‐nation referents) are heavily monitored and censured (Augoustinos & Every, 2007; Goodman, 2014). Billig (1988) argues that taboos around race and racism are grounded in implications of prejudice, which then indicates irrationality. Speakers then employ devices and practices that minimize those problematic implications (Augoustinos & Every, 2007). Van Dijk (1992) discusses the role of disclaimers (Hewitt & Stokes, 1975) such as ‘I'm not racist/prejudiced, but…’. In that, speakers orient to the possible recognisability of their utterances as problematic for their taboo nature (Augoustinos & Every, 2007) and deal with these possibilities (Goodman, 2014).

Relatedly, those reporting racism face the problem that their claims are rarely accepted (Essed, 1991b; Louw‐Potgieter, 1989; Rafaely, 2021). Those who are targets/victims of racism are at a disadvantage since their status as targets/victims comes in the way of receiving their claims and subsequent possibilities for justice (cf. Edwards, 2005b). Their accounts are suppressed or dismissed through practices that either offer alternative explanations for those events or challenge the characterisation of the event as racist. There is then a double bind in operation: much of racism takes place without making race/racism explicit, and complaints about racism are not accepted.

Yet another relevant facet here is the socio‐political context. Race researchers, who themselves are racialised in distinction to several authors above, show that the production of accounts about racism varies in different socio‐political contexts (Sambaraju, 2024a). Essed (1991a) points to notable differences in how racism is salient in reporting and accepting these reports between the United States and the Netherlands owing to differences in the ready availability of the history of enslavement in the former and the absence of such knowledge in the latter setting. Instead, knowledge about racialisation came about through their own experiences (see also Bonam et al., 2019; Salter, 2021). Notably, Essed (1991a) shows that the latter form of knowledge was treated as more contingent and open to doubt precisely because it comes from experience. Assertion and negotiation of rights to make claims about racism are deeply moral for the consequences of either suppressing and denying racism or suppressing the rights to make claims about racism (Condor et al., 2006). Doing so might be seen as ‘epistemic violence’ (Dotson, 2011). The salience of race categories for knowledge claims about racism is a useful line of inquiry in this regard (Durrheim et al., 2015; Windel et al., 2023). Ethnomethodological approaches have the potential to offer unique insights into how people themselves, in erstwhile colonised settings, can mobilise relevant categorisations in making claims about racism (Sambaraju & Minescu, 2019).

### Race categories and racial epistemics

Membership categorisation analysts examine the role of categories and categorisation practices in our social lives (Eglin & Hester, 1999; Sacks, 1992; Stokoe, 2012). For Sacks (1992), categories are ‘inference‐rich’ and make available common‐sense knowledge about persons so categorised in terms of their expected actions, rights, and entitlements. Membership categories themselves are organised by ‘which‐type’ sets called Membership Categorisation Devices (MCDs), such as race or sex, which specify the sort of inferences that can be legitimately made about the category member (Stokoe, 2012).

Sacks (1984) has argued that alongside the above inferences, there is a distribution of rights and entitlements to experiences and their telling along social categories and groups (cf. Bonam et al., 2019; Salter, 2021), to which Sacks tentatively ascribes intergroup suspicion and conflict. It *may be* expected that members in social categories who are routine targets of racial discrimination will be treated as having rights to racial knowledge more than members in social categories who are routine perpetrators of racial discrimination, owing to (expectations around) racial experiences (Sambaraju & Minescu, 2019). In that, their experiential knowledge (Heritage, 2012) can be treated as inviolate in making claims about racism. These mirror what Pomerantz (1984) has called Type I and Type II forms of knowledge (also see Kamio, 1994).

While experiential knowledge can be given epistemic primacy, it can be variously received and contested based on access to other epistemic resources (e.g., doctors can challenge nurses' claims based on observations) (see also Potter, 2012). Heritage and Raymond, across several papers (Heritage, 2012; Heritage & Raymond, 2012; Raymond & Heritage, 2006), have built upon earlier work by conversation analysts like Pomerantz (1984), Drew (1991), and Kamio (1997) to offer a systematic outline of the central role of knowledge‐claims in action formation and, consequently, for human sociality (but also see Lynch & Macbeth, 2016).

The territories of experience and knowledge, however, are differently orientated (Heritage, 2011). First, Heritage (2012) argues that territories of knowledge that persons have rights to, their *epistemic status*, are distinct from the *epistemic stance*, which is the interactional negotiation of their status. Speakers routinely attempt to maintain a congruence between their epistemic status and stance, meaning they wish to realise their rights and entitlements to certain knowledge in specific instances of interaction (Heritage, 2018). Those making claims about racism might make use of their category membership to draw upon experiences in ways to treat these as racist (Potter, 1996; Sambaraju & Minescu, 2019).

Second, while knowledge is transferable from a teller (K+) to a recipient (K−), one can neither transfer the *feel* of an experience nor is this expected (Heritage, 2011; Sacks, 1984). However, the experience can and is used to claim and enact rights and entitlements to knowledge (see Sacks' (1984) discussion of reporting a car accident or Potter (2012)). Telling of experiences similarly involves an epistemic gradient where the recipient is unaware of what the teller has seen, heard, felt, or experienced but has now received a report of what happened, was seen, heard, felt, or experienced as a specific event.

However, much of the research discussed above shows that reports about being targets of racism are not accepted. For instance, Zhang (2023) demonstrates two ways that reports about racism can be contested while considering that recipients cannot access the experience or details of the incident. Those contesting could either cast doubt on those claims through a reinterpretation of context or undermine these claims for lack of objectivity. It is then of interest to examine how issues of epistemic domains, rights, and entitlements, and their enactment, are negotiated in these interactions to claim that what has taken place is ‘racism’ (cf. Sambaraju & Minescu, 2019). In this paper, I focus on the salience of epistemic matters–experiential, specialised, or commonsense knowledge–in the making and receiving of claims about racism.

### The present study

I examine the above‐mentioned issues in accounts of anti‐Black racism in India in news media interactions. Indians and Black persons can be normatively associated with being targets of racial oppression. Racism and what it means for those experiencing it can be reasonably treated within the epistemic domains of those who are Indians and Black persons in ways that negotiate whether instances of violence are racist (Sambaraju, 2021). However, in instances where the perpetrators are Indian, there are likely to be issues of stake and interest in readily accepting anti‐Black racism in India (Potter, 1996).

Black Africans in India report pervasive discrimination verbally and physically, or, more societally, in the form of limited access to housing and employment (Rathi, 2017). This has taken a more serious turn, at least since reports of increasing numbers of violent physical attacks began in 2012 (which curiously died off after 2017). These include a series of lynching incidents of Black African persons in Goa, New Delhi, Hyderabad, and Bengaluru. Further reports include the death of a Congolese person in New Delhi in 2016, a Nigerian national in Goa in 2013, and the death of a Burundian national in Chandigarh in 2014 after an attack in 2012 (Venkatraman, 2016).

Black Africans, however, claim that racism and discrimination are not accepted as relevant concerns by authorities (Rathi, 2017). In news media programs, Black peoples' accounts were treated as testimonials in ascertaining and establishing the relevance of racism in India, where Black people offered descriptions of their experiences in ways that attend to the broader aspect of possibilities for racism in India and more contingent aspects of how accounts are produced as relevant or veridical accounts about racist incidents. In these instances, the concern is how to orient and manage Black persons' expertise in identifying, knowing, and claiming racism in India. I then examine how rights and entitlements to racial knowledge are treated as distributed along social categories and used in negotiating claims about racism.

## METHOD

### Data and participants

The data for this study are transcripts of broadcast news media program videos collected over the Google Video Archive Search Engine using the search string ‘Africans + Attacks + India + debate or discussion’ for this study, the period between years 2012 to 2017 (inclusive)^1^. Selecting for non‐repetitive debates, without any language restrictions that were broadcast for a national or international (rather than regional) audience, where the videos included both contributions of the host or news anchor and panellists, and where the content of the discussions was indeed relevant to racism in India, yielded 16 videos^2^. Each of these programs lasted more than 30 min; 4 of these were conducted in Hindi‐Urdu, and the rest were in English (12) (see Appendix A).

These videos were accessed either on YouTube or news agency websites and watched repeatedly to gain an intimate familiarity with the topics and issues being discussed. The videos involved talk about various *topics* such as whether these acts or Indians can be appropriately categorised as racist (see Sambaraju, 2021), the origins of racism in India, experiences of Black peoples, the role of authorities, and possibilities for addressing these issues. I focused on those instances where Black persons were invited to inform the prevalence and understanding of anti‐Black racism in India by the interviewers. These instances were transcribed using the modified Jeffersonian system to enable a close analysis, whereas other parts were transcribed merely verbatim (Jefferson, 2004).

### Analytic procedure

The transcripts were analysed using procedures of discursive psychology (Edwards, 2005a; Potter, 2021). In line with the arguments of discursive psychologists, speakers' personal experiences or other accounts of racism are examined for how it is that these are produced, taken up, and treated as attending to interactional tasks and concerns (Potter, 2012). The analysis also employed certain conceptual tools from conversation analysis, such as those that deal with negotiating rights to or accessing relevant knowledge about racism in India.

Conversation analysts argue that turns‐at‐talk routinely attend to epistemic differences for the speakers involved (Drew, 2012; Heritage, 2012). In the present instance, several concerns coincide: first, as news discussions, epistemic differences between participants and the audience are built into the interaction (Brinkmann, 2007; Heritage, 2018). Talk is likely to proceed in ways to generate ‘new’ information. Second, interviewees are likely to be treated as in possession of unique experiential knowledge that only they have access to, but in ways to implicate that the interviewers are possibly aware of this form of knowledge: a possibly racist incident. Third, interviewers, other panel members, and audiences might not have experienced racism or in a way that is relevant for the ongoing interactions; they are likely to make claims about racism as a generic societal phenomenon–‘racial common sense’. Fourth, participants here can make claims to varying levels of familiarity with India and goings‐on in India in ways to affirm or undermine claims about racism in India. Experiential accounts are relevant as these can be used as evidence to make claims about racism in the wider community or, in this case, India.

Given the above considerations, the analysis examined how speakers orientated to, claimed, enacted, and negotiated their rights to knowledge about racism in India in instances where the interactions focused on accounts of anti‐Black racism in India.

### Positionality statement

The descriptions and categories that participants and I use will necessarily contribute to specific versions and views about the events and/or the wider context. I am a person of colour but am not Black. I am from India and live in Europe/the United Kingdom. I am intimately familiar with certain forms of contemporary and historical racism. For the present case, however, I am in a ‘majority’ position in contrast to racism in Euro‐American contexts where I'm minoritized. As a result, I'm in a unique position to ‘know’ racism as both a possible target and a perpetrator. The present study is, in part, motivated by this. The aim here has been to examine how such possible entitlements and positions are implicated in interactions where race and racism are topical and used to negotiate interactional concerns over racism.

## RESULTS

Across the data examined here, the interviewers invite Black persons to talk about racism by relaying their experiences, views, and perspectives on racism in India. Black persons are treated as *uniquely* entitled to racial knowledge, which the interviewers, other participants, or the overhearing audiences are not (Heritage, 2011; Sacks, 1984). Results show speakers could develop distinct forms of epistemic gradients, based not only on racial category membership but also on access and entitlements to common sense knowledge, specialist knowledge about non‐racial matters, or alternative access to racial matters. These epistemic entitlements were deployed to attend to three forms of collaborative race‐talk as social action (Condor et al., 2006): establishing racism as a concern for Black persons in India, confirming or correcting more widely held views about anti‐Black racism, and challenging claims about racism.

### Establishing anti‐Black racism in India

In Extracts 1 and 2, interviewees' experiences of racism in India are explicitly treated as indicating that their knowledge about racism is different from the interviewer's, the audience's, and unspecified others. While in Extract 1, the interviewee directly draws from their experiences to take up and enact such epistemic entitlements, in Extract 2, the interviewer marks the possibility that the interviewee has experiential knowledge.

In both extracts, interviewees are invited to talk about racism, and while doing so, their accounts are treated as insufficient to establish racism. Interviewers participate in the production of descriptions of interviewees' experiences in ways to claim for themselves mundane knowledge that is commonly available for those who can be seen as a ‘layperson’ while leaving out the more pertinent matters of ‘expertise’ in racism to the interviewees (cf. Kitzinger & Mandelbaum, 2013). This allows for maintaining congruence between entitlements and the enactments of the epistemic territories of the interviewees regarding establishing what is racism (Heritage, 2018). However, there is no effort either by the interviewee or the interviewer to suppress or deny racism as is frequently found in Euro‐American and other settler colonial settings (Goodman, 2014).

Extract 1 comes from the video transcript of an episode titled ‘We The Racists? India's ‘Unfair’ Obsession, Skin‐Deep Prejudice’, published on April 9, 2017, by New Delhi Television. This episode involved a female interviewer talking to a mix of ‘experts’ like police officials, politicians, academics, and laypersons. The talk below comes after an account from a female politician on how racism in India is an outcome of skin‐colour‐based prejudice. The interviewee (Eddy) is a male African American who was in the show with his Indian wife.

#### Extract 1

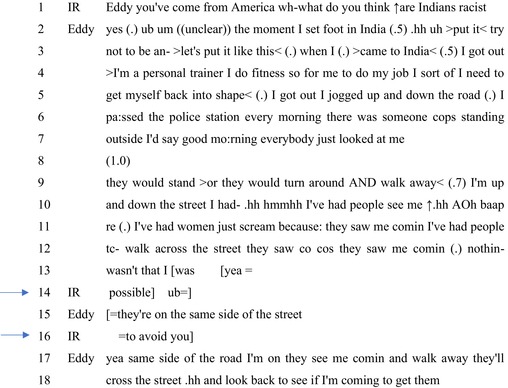

Of interest are the interviewer's interjections, framed as interrogatives (Heritage, 2013), at lines 14 and 16, which come while the interviewee is giving experiential accounts about racism in India. This account is being given in response to the question about whether Indians are racist, which does not explicitly foreground Eddy's experiences as a possible target of discrimination in India.

In using the preface that categorises Eddy as somebody who has ‘come from America’ (line 1) (see Schegloff, 2007a), it implicates specific forms of knowledge about racism for Eddy, which can be used to respond to the question. Eddy is visibly Black (cf. Nishizaka, 2021) and is treated as having an entitlement to knowledge about racism. The question then sets up for Eddy an account that stems from such a K+ position. Starting as an opinion‐seeking question–‘wh‐ what do you think’–to a fully formed polar question (Raymond, 2003): ‘↑are Indians racist?’ (line 1). While in its grammar it sets up ‘yes’ as a response, a ready agreement might be problematic given not only the context of this interaction but also because it could come across as making a highly unfavourable and categorical moral judgement (Condor et al., 2006).

In response, Eddy offers a type‐conforming ‘yes’ (line 2) (Clayman & Loeb, 2018) followed by a detailed account involving several types of experiences in India. Eddy then treats his experiential knowledge as grounds for having agreed that Indians are racist. Eddy's response does orient to the problematic nature of his account, in so far as this can be heard as condemning India, which is that this response avoids other ways of responding–‘try not to be an‐’ (lines 2–3). For instance, his initial attempt–‘the moment I set foot in India’ (line 2)–is hearable as initiating an account that could offer damning evaluations of India and Indians. Eddy shifts his footing (Goffman, 1981) to indicate that the account is being delivered in consideration of himself and others (Condor et al., 2006): ‘Let's put it like this’ (line 3).

The oncoming account expectedly offers several details (Potter, 1996), inclusive of his and others' actions: he ‘got out and jogged up and down the road’ (line 5) and greeted, while others would ‘just look[ed]’ at him, ‘stand’, or ‘turn around AND walk away’ (line 9). First, these descriptions treat him as engaged in unproblematic actions: as a ‘personal trainer’, it is unsurprising that Eddy goes jogging (Sacks, 1992). Further, the three‐part listing (Jefferson, 1990) of others' responses (‘just looked’, ‘stand’, and ‘turn around AND walk away’) indicates a commonality of not returning a greeting. Second, these present him as initiating the first‐pair part of the ‘greeting‐greeting’ adjacency pair (Sacks, 1992), where the absence of a relevant second‐pair part from those others is treated and hearable as problematic. While such responses to his actions could be read as being rude, ignorant, or culturally less salient, his descriptions that these transgressions were dispositions (Edwards, 2005b)–‘they would’ (line 9) (also see Palmer, 2001)–allow for inferring the problematic aspect: something about him that facilitated these problematic responses.

Subsequently, he offers an upgraded description of his experiences in another three‐part list (Jefferson, 1990) where each item is prefaced with ‘I've had’ (lines 10 and 11). This preface presents each incident as having taken place multiple times and offers the inference that these forms of reactions to his presence on the street are routine for him, as a Black person or as someone new to India. These items also allow for inferences that he is indeed subject to reactions and responses from Indian people that are inexplicable: intentional or contingent. Eddy himself orients to such possibilities as seen in his mark of exasperation–‘.hh hmmhh’ (line 10) –at the beginning of this set of descriptions about his walking on a street in India.

The relevance of this is in establishing whether what Eddy has experienced is indeed racism. At lines 12–13, Eddy proceeds to reject possible alternatives–‘nothin‐ wasn't that I was’–which is interrupted by the interviewer. The extreme‐case formulated response (Pomerantz, 1986) suggests that no explanation is salient, but that Eddy is Black. At lines 14–17, the interviewer offers one possibility where other passersby could not ‘avoid’ Eddy unless they crossed the street; that is, she introduces possible contingencies. This involves using mundane or routine knowledge of when people might cross the street: when there is not enough room for other parties. The interviewer's explanation stems from her position as a ‘layperson’ rather than as someone who can make claims about racism in India or about Eddy's personal experiences. In that, the interviewer treats a personal experience as subject to normative understandings of what someone walking on a street *might* experience (cf. Heritage, 2011). The interviewer positions herself as in a position of lesser epistemic entitlement relative to Eddy, maintaining an epistemic gradient while managing to indicate that what Eddy had been subject to can only be explained as racism. Eddy confirms that both parties could have kept walking on the same side of the street but did not. This allows for making salient that others' responses to Eddy stem not from possible contingencies but are intentional and so racist.

In distinction to Extract 1, in Extract 2 the interaction is more explicitly about experiences of racism. The interviewer invited Black persons to talk about racism to demonstrate to the broadcast audience the prevalence and occurrence of anti‐Black racism in India. The interviewer then clearly outlines distinct territories not only of experience but also of rights and entitlements to make claims about racism (cf. Heritage, 2011).

In Extract 2, the interviewers' contribution, again indicating a layperson's knowledge, works to establish racism as salient for the interviewees' account while maintaining an epistemic gradient that reserves epistemic rights and access with Black persons. Extract 2 comes from a video transcript of a program titled ‘Attacks on Africans: Students speak out’ published on May 25, 2016, by New Delhi Television. Here the interviewer was joined by several Black persons introduced as ‘students’ in an off‐studio location alongside panellists in the television studio.

#### Extract 2

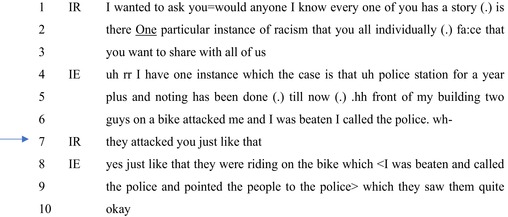

Of interest here is the interviewer's ‘declarative question’ (Heritage, 2013) at line 7: ‘they attacked you just like that’. This comes while the interviewee is narrating an incident of facing physical violence, in response to the interviewer's invitation to narrate an ‘instance of racism’ (line 2). Heritage (2018) notes that recipient design is the ‘most wide‐ranging and significant’ (p. 19) aspect of navigating epistemic territories. The question design here is noteworthy for setting up interviewee responses not as those which will transmit experiential aspects to the interviewer or others, but to accomplish a practical task in a broadcast show (Heritage, 2011; Sacks, 1984).

First, the interviewer shifts the trajectory of the question (‘I wanted to ask you’ (line 1)), and the interaction to follow, by repairing (Schegloff, 2007b) the question from indicating *possibilities* of experiencing racism to asserting that potential interviewees would have had such experiences: ‘I know every one of you has a story’ (line 1). Second, the interviewees' category membership as Black persons is treated to implicate having been targets of racism through the use of the extreme‐case formulation (Pomerantz, 1986) ‘every one’ (line 1). Further, the characterisation–‘has a story’–is suggestive of owning information about being targets of racism, which is to be conveyed to the audiences.

Third, the design of the question is noteworthy in managing the stake of the interviewer (Potter, 1996). The question, framed as an interrogative, ‘is there’ (lines 1–2), is less directed at ascertaining whether they are targets of racism and more at inviting them to share their stories. Notable also is the extreme case formulated (Pomerantz, 1986) description of the recipients: ‘all of us’ (line 3). This minimises her vested interest in interviewees' racial experiences and makes salient the role of a broadcast audience, who are positioned as not knowing about racism in India.

The interviewer then outlines distinct territories of and access to knowledge for those who will provide recognisable instances of racism, those who know that this set of Black persons in the studio has experienced racism, and the audiences who are to be informed about anti‐Black racism in India (cf. Rathi, 2017).

The interviewee at line 4 self‐selects themselves: ‘I have one instance’ (Clift, 2016). However, there is an orientation to possible trouble in doing so–‘uh r r’ (line 4)–indicating that the oncoming account might not satisfy the task set up by the interviewer. The account starts with a description of how the incident was taken up (or not) by the authorities: ‘police station for a year plus and nothing has been done (.) till now’ (lines 4–5). This complaint indicates that normative duties expected of police personnel were not carried out (cf. Sambaraju, 2021; see also Extract 1) allowing for the inference that membership in certain racial categories might involve alternative forms of responses from the police (cf. Shrikant & Sambaraju, 2021).

The relevance of this is that the interviewer, although narrating an instance of racism, has not made it explicit: ‘In front of my building, two guys on a bike attacked me, and I was <beaten. I called the police’ (lines 5–6). The interviewer similarly orients to this as incomplete given the tasks set up by her invitation. Her interrogative (see Heritage, 2013) at line 7–‘they attacked you just like that’ (line 7)–points to the absence of an explicit reason for the violence, like that which involves robbery or a mutually aggressive episode (Kirkwood et al., 2013).

In making explicit that there was neither another reason for the violence nor that it was provoked by the interviewee, the interviewer allows for the inference that the violence was possibly racially motivated. The interviewer implicates herself as entitled to ‘layperson’ knowledge about how violent attacks take place and specifically about the possible reasons for it (Kitzinger & Mandelbaum, 2013). Notably, the interviewer does not take as the issue that the police did not respond acceptably. It is instead the reason for the violence, which is perhaps a more easily recognisable aspect of racism. The interviewee confirms that there was no explicit reason for the violence and proceeds to point to issues with the police, which are not pursued further.

In Extracts 1 and 2, while interviewees are treated as in possession of knowledge about racism (through their experiences, for instance), the interviewers treat the interviewees' accounts as insufficient to establish anti‐Black racism in India in news programs, without the involvement of the interviewer (interviewees reject alternative explanations). Interviewers' involvement as persons with regular or commonplace knowledge about the goings‐on, at times, voiced for the overhearing audiences, allows for making clear that the interviewees' experiences can be seen as racial. Below are two extracts where interviewee accounts are treated as sufficient.

### Confirming anti‐Black racism in India

In Extracts 3 and 4, the interviewers' questions explicitly treat the interviewees as those who have relevant experiential knowledge to make claims about racism in India. The interviewees proceed to offer such responses.

Extract 3 comes from the video transcript of a news program conducted by the news agency India Today, titled ‘To The Point: Are Indians Racist?’ published on May 30, 2016. Thapar invites Mobolade Jonathan to offer his account after accounts by other guests in the show.

#### Extract 3

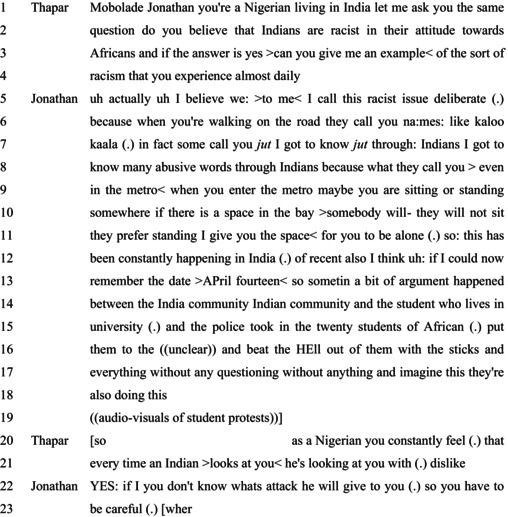

Thapar's first question to Jonathan is presented as following from a similar question posed to an unspecified person. This is in a ‘yes/no’ interrogative polar question format–‘do you believe’–with a preference for ‘yes’ (Raymond, 2003), which Thapar himself supplies to lead to the second question. The K+ status ascribed to Jonathan is specified as a state of ‘belief’, suggesting possibilities for doubt or evidential confirmation. It is here that Thapar offers the second question as a tag to the first question (Hayano, 2012), which foregrounds the possibility that Jonathan has experienced racism, by virtue of his category incumbency, and treats him as in the possession of information about racism in India in ways to make claims about Indians as racist or not.

Jonathan orients to his epistemic entitlements to make claims about racism in India as arising from his experiences of racial discrimination: ‘I got to know many abusive words through Indians because of what they call you’ (lines 7–8). In doing this, Jonathan treats his claims as grounded in inviolate sources of knowledge (Sacks, 1984). This allows Jonathan to downplay possible inferences that his ongoing account is merely tied to his category incumbency and addresses his stake in producing an account that would suggest the prevalence of racism in India (Potter, 1996). Jonathan characterises these forms of racism as ‘deliberate’, in opposition to possible characterisations of unfavourable acts as accidental. In addition to instances of name‐calling, Jonathan describes other forms of racism: one which is generic, during daily travel, and another which is specific to a particular incident.

The first refers to racial abuse in mundane social settings such as ‘walking on the road’ (line 6) and ‘in the metro’ (line 9). Jonathan's descriptions include relevant detail (Potter, 1996) in terms of the specifics of the racist actions, such as the racial slurs used, which build up the facticity of the account. In settings of public transport, Jonathan's descriptions offer the inference that fellow travellers dislike individuals like himself: ‘if there is a space in the bay, >somebody will‐ they will not sit; they prefer standing. I give you the space< for you to be alone’ (lines 11–12). Notable here is that the interviewer could have interjected in a similar fashion to Extract 1 and suggested alternative explanations. However, this is not done, indicating that Thapar treats Jonathan as offering relevant descriptions that confirm anti‐Black racism in India. The second instance relates to possible institutional racism when ‘the police’ severely attacked a group of ‘African’ (line 15) students without due process (cf. Extract 1) (Sambaraju, 2021). While Jonathan is positioned as an expert in anti‐Black racism in India for his category membership–‘Nigerian living in India’–his descriptions treat his knowledge as an outcome of his experiences.

Thapar treats these detailed descriptions as indicative of pervasive anti‐Black racism in India experienced by Jonathan as an incumbent in the category ‘Nigerian’ at the hand of those in the other category ‘Indian’: ‘as a Nigerian you constantly feel’ and ‘every time an Indian >looks at you< he's looking at you with (.) dislike’ (cf. Durrheim et al., 2015; Sambaraju, 2021). The extreme case formulation (Pomerantz, 1986) (‘every time’) works to treat racism as normatively bound to category membership. Thapar then treats Jonathan's account as salient in making broader claims about racism in India.

In Extract 4, the interviewee offers descriptions of anti‐Black racism based not merely on personal experience to confirm or correct others' assumptions. Extract 4 comes from a program titled ‘Are Indians Racist’ on the series ‘The Big Fight’, hosted by Vikram Chandra and published in February 2013 by New Delhi Television. The talk below comes after several other guests on the show offered their accounts of racism in India.

#### Extract 4

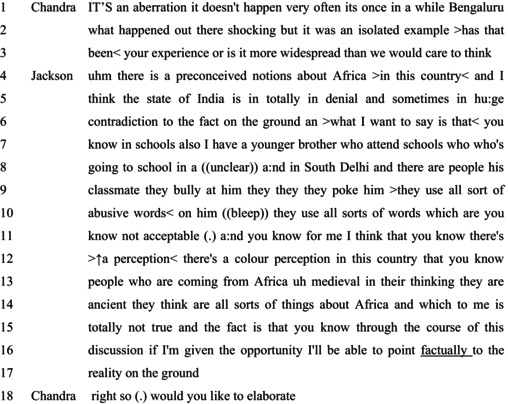

Chandra treats Jackson as in a position to confirm or challenge other claims about racism in India through his experiences (cf. Kitzinger & Mandelbaum, 2013). These other claims are developed as indicating that racism in India is rare, in a three‐part list format (Jefferson, 1990): ‘It's an aberration’, ‘it doesn't happen very often’, and ‘it's once in a while’. Chandra treats Jackson as possibly in a position to counter this view, based on his experiences: ‘Has that been your experience, or is it more widespread than we would care to think?’ (lines 2–3). Through this question design, Chandra develops an epistemic gradient where Jackson's experiential knowledge can correct and inform Indians' (‘we’) views on anti‐Black racism in India (Rathi, 2017).

In response, Jackson frames his account as that which will counter the view voiced by Chandra (Goffman, 1981), without, however, making it explicit that the account to be given is experiential. Instead, Jackson is in an epistemically privileged position to counter prevalent views about ‘Africa’ as ungrounded in facts–‘preconceived notions’ (line 4). These are made explicit towards the end of his account at lines 13–14, in another three‐part list format (Jefferson, 1990): ‘people coming from Africa are mediaeval in their thinking’, ‘they are ancient’, and ‘they think all sorts of things about Africa’. He explicitly treats this latter set of views as incorrect (‘totally not true’ (line 15)) and himself as able to correct these views by providing facts and the ‘reality on the ground’ (line 17). Jackson describes two types of incidents, neither of which involve him personally.

First, Jackson describes incidents at ‘schools’ (line 6) in ways that treat racism as pervasive: ‘in schools also’. He establishes his access to such incidents–‘younger brother’ (line 7)–and offers relevant details that establish the veridical nature of his account (Sambaraju & Minescu, 2019). He recruits the common knowledge of Chandra, co‐present panellists, and the overhearing audience by characterising the verbal abuse as of a type that is not part of interactions among school‐going children to indicate possible racism: ‘words which are, you know, not acceptable’ (line 10).

Second, he offers generic descriptions of how persons from Africa are viewed: ‘colour perception in this country’ (line 12). Jackson indicates that offering these descriptions as part of the response is expected of him, through offering views on racism from a personal footing (Goffman, 1981): ‘and you know, for me, I think that’ (lines 10–11). Jackson then offers a ‘his‐side’ account that treats him as in possession of relevant knowledge about racism while not offering claims based on his personal experience. Chandra at line 18 accepts this status of Jackson and invites further descriptions, without indicating that Jackson's task is complete, as Thapar does in Extract 3.

In distinction to Extracts 1 and 2, in Extracts 3 and 4 the interviewers present to the viewers that the type of knowledge that the interviewees possess is particularly relevant to confirm or correct others' views about anti‐Black racism. This is facilitated not only by the normative understanding that interviewees as targets/victims possess such experiential knowledge but also by the interactional foregrounding of their epistemic status (Ekström, 2007). Below we see instances where the relative difference in epistemic territories about racism in India is negotiated to challenge claims about racism.

### Challenging anti‐Black racism in India

In Extract 5, the interviewer uses two forms of knowledge: routine or mundane and specific to her being a Hindi language user. These different uses affect alternative forms of account production: the first to affirm the problematic nature of the event and the second to complicate the inference that the event is racist. Extract 5 comes from the same video as in Extract 2 and comes immediately after the talk shown there.

#### Extract 5

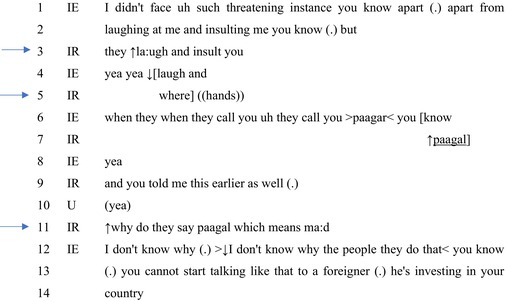

The interviewee's account is a ‘second story’ (Sacks, 1992) as it comes after the account examined in Extract 2, and he orients to his account as relating to the topic of narrating racist incidents. His incident, however, is downgraded relative to that of the interviewee in Extract 2, indicating a lower stake in producing this account: ‘didn't face uh such threatening instance’ (line 1). The interviewee's relatively downgraded instance is that of having been laughed at and insulted. Following the micropause, the interviewee prepares to expand his account in ways that might lead to alternative inferences: ‘(.) but’ (line 2). The interviewer, however, interrupts and treats his account as somehow inadequate and therefore repairable in ways to offer further information through the repeat formulation (Robinson & Kevoe‐Feldman, 2010): ‘They laugh and insult you’ (line 3). The interviewer then indicates that the task set for the interviewee is incomplete and that more information is needed.

The interviewee goes on to offer an account that initially confirms–‘yea yea’ (line 4)–and then explicates this further at line 6, overlapping with the interviewer's directions for a specific form of explication: ‘where’. Thus far, the interviewer uses routine or mundane knowledge about experiences to treat such experiences as problematic.

The interviewee describes instances of being laughed at and insulted when unspecified ‘they’ engage in name‐calling: ‘call you >paagar<’ (line 6). The interviewer's repeat formulation this time selects the term ‘paagal’ as perhaps in need of repair via further explication (Robinson & Kevoe‐Feldman, 2010), to which the interviewee offers a confirmation. Subsequently, the interviewer herself offers further evidence of the use of this term in referring to Black persons through recruiting the report of another member: ‘And you told me this earlier as well’ (line 9).

The interviewer combines both sources of experiential evidence to treat this as somehow inexplicable as a racist utterance by offering one translation of the term: ‘which means ma:d’ (line 11). The translation involves using her knowledge of Hindi‐Urdu and the standard meaning of that term in Hindi‐Urdu. This question then treats her as not having access to relevant information about the use of the term in ways that might constitute racism. While the interviewee avows a lack of knowledge in ways to manage their stake in explicating why others could be taunting them, he treats it as insulting and develops racism as an explication by contextualising its use against ‘foreigner[s]’ (line 13).

In Extract 6, entitlements to making claims about racism based on personal experiences are undermined and subsequently remade in other ways. Extract 6 comes from a video titled ‘Are Indians the most racist?’ published by NDTV on June 27, 2009. The interviewer, Nidhi Razdan, is holding a panel discussion with participants Ravi Shankar Prasad, Madhu Kishwar, and Malena Amusa, a female African American then working as a journalist in India.

#### Extract 6

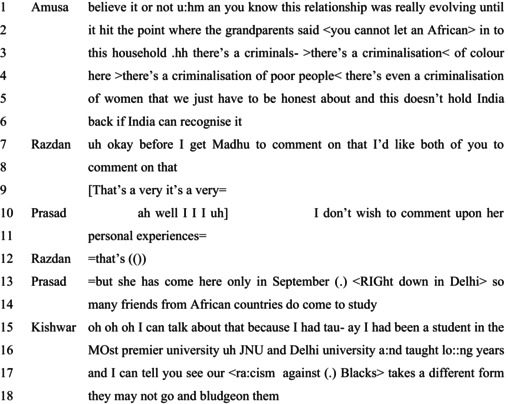

Amusa here is describing an instance of personal experience of discrimination in her romantic relationship based on her ethnicity: ‘<You cannot let an African> into this household’ (line 2). Razdan's uptake of this account shows that Amusa's account fits with the expectations of demonstrating racism as is seen in the incomplete but upgraded assessment by Razdan (Alroumi & Lahlali, 2022; Lindström & Mondada, 2009): ‘That's a very, it's a very’ (line 9). Razdan also invites other panellists to ‘comment’ on Amusa's account of personal experiences of racism.

What follows then are remarks from two panellists: Prasad and Kishwar. First, Prasad resists a ready alignment with the upshot of Amusa's account. Rather, he formulates Amusa's account as describing ‘personal experiences’ in ways that limit his rights to remark upon these. Prasad treats Amusa as rightfully entitled to her experiences, without, however, endorsing (or rejecting) the claim that these experiences constitute racism (cf. Heritage, 2011; Sacks, 1984).

Rather, at lines 13–14, Prasad undermines the relevance of Amusa's experiences for making broader claims about racism in India in two ways. First, he points to the limits of her knowledge about India given certain temporal and geographical constraints: ‘She came here only in September’ (line 13) and ‘down in Delhi’ (line 13). Without undermining her rights to make claims that she experiences racism, he raises questions about her access to events and details that might enable her to make such claims. In that, Prasad develops a distinction between having experiences and epistemic entitlements based on experiences.

Second, Prasad builds up his epistemic stance through claims to his access to possible racial interactions: ‘So many friends from African countries do come to study’. His reference to ‘friends’ indicates his entitlement to knowledge about racism against Africans in India (cf. Sambaraju & Minescu, 2019). Together with the claim about several persons coming to India, Prasad suggests that Amusa's experiences have limited importance for discussions on racism in India, without challenging that she experienced racism.

Kishwar's response, however, counters the inference offered by Prasad while attending to the type of entitlements that Prasad claims for himself. Kishwar makes explicit her entitlement to speak about students from countries in Africa: ‘I can talk about that because I had been a student at the most premier university, uh, JNU and Delhi university and taught long years’ (lines 15–17). Of note is the repeated use of the news receipt particle (Heritage, 1998) ‘oh’ at the start of her account, indicating an acknowledgement that Prasad's treatment of Amusa's epistemic stance is relevant to making claims about racism in India.

While Prasad and Kishwar both make claims about anti‐Black racism in India, they do so by establishing their entitlement to access such instances through others, either ‘friends from African countries’ or Black students in Delhi. Both Prasad and Kishwar then develop varying forms of epistemic gradients concerning Amusa and the prospective audiences in offering differing versions of anti‐Black racism in India. Claims about racism can then be challenged on grounds of possibilities for knowing and having entitlements to knowledge about racism.

## DISCUSSION

The above analyses contribute to extant scholarship on the social psychology of racism as it pertains to narrating or reporting racism (Sambaraju & McVittie, 2021). It shows that an important aspect of what others have called the collaborative production of race‐talk (Condor et al., 2006) is that of negotiating epistemic gradients. Co‐present interlocutors, although not in the position to claim similar experiences or undermine the experiences of those talking about racism, have at their disposal other forms of knowledge and entitlements to these, which impact the production of race talk. The paper has then shown how some features of individuals, like category membership, cultural/linguistic familiarity, or common‐sense notions, are implicated in the production of claims about racism.

In the extracts analysed above, speakers developed and negotiated distinct epistemic gradients in accomplishing specific actions around establishing, confirming, and challenging claims about racism in India. First, interlocutors used common‐sense knowledge about daily occurrences in collaboratively establishing the prevalence of racism. Second, interlocutors outlined distinct epistemic territories of knowledge based on the experiences of Black persons in India to confirm anti‐Black racism in India. Third, interlocutors claimed other forms of epistemic entitlements to complicate or challenge claims about racism. Across these actions, speakers orientated to the salience of epistemic territories, bound to category membership or otherwise, and their realisation in interactions involving race talk.

The findings further current knowledge about race‐talk by showing that one reason why reporting or talking about racism is complex is because of the varied expectations about what counts as racism (Essed, 1991b; Louw‐Potgieter, 1989; Zhang, 2023). For speakers here, Black persons were treated not only as uniquely entitled to experiences of racism in India but also in the position to make claims about racism and its features based on their experiences (Sacks, 1984; Sambaraju & McVittie, 2021; Sambaraju & Minescu, 2019). However, this entitlement was variously negotiated: affirmed, attuned, and supported, or complicated and challenged. In that, co‐present interlocutors are implicated in a foundational manner: their entitlements to knowledge about mundane socio‐cultural life, contrasting suppression of entitlements to knowing about racism, or entitlements to other forms of knowledge are implicated in how claims and reports about racism are received.

Social constructionist approaches to racism must then consider how epistemic issues are intertwined in negotiations of what is to count as racism. To claim that an event or action was racist, it is not only sufficient that a target claims so, as supported by previous research (Essed, 1991b; Louw‐Potgieter, 1989), but that the description of the event needs to be made in specific ways. This latter production implicates various forms of socially distributed knowledge positions of interlocutors (Heritage, 2012). The social construction of racism (and race) implicates wider aspects of social life alongside those of colonialism, segregation, and other forms of racialised oppression (Sambaraju, 2024b).

These findings are noteworthy in light of another foundational aspect of social interaction: recipient design (Heritage, 2013; Schegloff, 2007a). Not only are reports or complaints about racism delivered in ways that attend to their reception, but what we see here is the role of the interviewers in shaping the form of response that the interviewees are to offer. In that, Black persons' reports or claims about racism attended not only to how these would be received but also to how these are set up as responses in light of specific epistemic territories and their realization. These findings then suggest that perhaps it is not just the reporting practices of targets of racism that need to be examined and acted upon, but also the practices and broader structures that are set up to receive reports and complaints about racism.

The data are from a specific context and involve groups that are not routinely the focus of examination in social or discursive psychology. For this reason, the salience of these findings will be questioned. The attempt here has been to treat interactions and phenomena in a research‐neglected part of the world as just as important as Euro‐America. What is noteworthy, perhaps as a feature of this setting, is that racism is not directly suppressed or denied (but see Extract 6) as is a routine feature of research from within Euro‐American settings (Sambaraju, 2024a).

To address the concern mentioned earlier in this paper, which was about issues in reporting and denials of racism, the present findings suggest that one aspect that should be attended to is how epistemic territories that are normatively bound to targets are realised in the interaction. Not only do those receiving reports about racism need to be careful and watch out for tellers' efforts at relaying racism, but also how it is that those relaying these reports are treated regarding their relation with what is racism. Individuals' identities and category memberships are of significance in reporting and receiving reports about racism.

## AUTHOR CONTRIBUTIONS

**Rahul Sambaraju:** Investigation; funding acquisition; conceptualization; writing – original draft; writing – review and editing; methodology; validation; visualization.

## CONFLICT OF INTEREST STATEMENT

The author declares no conflict of interest in the publication of this manuscript.

## Data Availability

The data that support the findings of this study are available in YouTube at https://www.youtube.com/. These data were derived from the following resources available in the public domain: – YouTube, https://www.youtube.com/.

## Footnotes

^1^
Possibly reflecting how news cycles work, after 2017, attacks on Black persons or Africans were barely seen in broadcast news. To limit possibilities for sparse or extremely heightened news coverage to skew results, I have chosen to limit the data for the years 2012–2017.

^2^
The search was conducted using a Google Chrome browser that is attached to my account. Results for others and from other browsers are likely to vary.

## A.1 Data sources

**Table:**

Extract	Video source/title	Link
1	NDTV: Attacks on Africans: Students speak out	https://www.youtube.com/watch?v=gUsL‐zn1SI8
2	NDTV: We The Racists? India's ‘Unfair’ Obsession, Skin‐Deep Prejudice	https://www.youtube.com/watch?v=liG2o4pP6ko
3	India Today: ‘To The Point: Are Indians Racist?’	https://www.youtube.com/watch?v=OJcxCMVFxWk
4	NDTV: The Big Fight: Are Indians racist?	https://www.youtube.com/watch?v=OCcgx16e8DQ
5	NDTV: Attacks on Africans: Students speak out	https://www.youtube.com/watch?v=gUsL‐zn1SI8
6	NDTV: Are Indians the most racist	Are Indians the most racist? (ndtv.com)
